# Anti-inflammatory polymer electrodes for glial scar treatment: bringing the conceptual idea to future results

**DOI:** 10.3389/fneng.2014.00009

**Published:** 2014-05-13

**Authors:** Maria Asplund, Christian Boehler, Thomas Stieglitz

**Affiliations:** ^1^Biomedical Microtechnology, IMTEK, Albert-Ludwigs UniversitätFreiburg, Germany; ^2^Freiburg Institute for Advanced Studies FRIAS, Albert-Ludwigs UniversitätFreiburg, Germany; ^3^BrainLinks-BrainTools Cluster of Excellence, Albert-Ludwigs UniversitätFreiburg, Germany

**Keywords:** conducting polymer, dexamethasone, drug delivery, glial scarring, neural interfaces

## Abstract

Conducting polymer films offer a convenient route for the functionalization of implantable microelectrodes without compromising their performance as excellent recording units. A micron thick coating, deposited on the surface of a regular metallic electrode, can elute anti-inflammatory drugs for the treatment of glial scarring as well as growth factors for the support of surrounding neurons. Electro-activation of the polymer drives the release of the substance and should ideally provide a reliable method for controlling quantity and timing of release. Driving signals in the form of a constant potential (CP), a slow redox sweep or a fast pulse are all represented in literature. Few studies present such release *in vivo* from actual recording and stimulating microelectronic devices. It is essential to bridge the gap between studies based on release *in vitro,* and the intended application, which would mean release into living and highly delicate tissue. In the biological setting, signals are limited both by available electronics and by the biological safety. Driving signals must not be harmful to tissue and also not activate the tissue in an uncontrolled manner. This review aims at shedding more light on how to select appropriate driving parameters for the polymer electrodes for the *in vivo* setting. It brings together information regarding activation thresholds for neurons, as well as injury thresholds, and puts this into context with what is known about efficient driving of release from conducting polymer films.

## Introduction

Recent advances in the field of conducting polymers point out their potential as drug delivery coatings from the surfaces of microelectrodes. This is of interest considering it comprises an opportunity to target cells in the close vicinity of an implant with high spatial and temporal control of release. Glial scarring is a physiological process that deteriorates electrode function by forming a substantial barrier for signal transduction. Persistent inflammation, following the scarring process, is believed to be the reason why neurons are lost at the site of the implant further complicating high resolution in recording and stimulation (Turner et al., 1999; Szarowski et al., 2003; Biran et al., 2005). Systemic treatment using anti-inflammatory drugs such as Dexamethasone (Dex) has been suggested as a possible strategy for facilitating close integration of the implant with neural tissue (Spataro et al., 2005). Conducting polymer electrodes designed to elute drugs upon electro-activation are an alternative to systemic treatment of glial scarring (Abidian et al., 2006; Wadhwa et al., 2006; Evans et al., 2009; Richardson et al., 2009; Luo and Cui, 2009a,b; Stevenson et al., 2010; Yue et al., 2013). Polymers in question are mainly Polypyrrole (PPy), poly(3,4-ethylene dioxythiophene) (PEDOT) but more recently also polyterthiophene (PTTh) has been suggested as a candidate (Stevenson et al., 2010). This functionality is until now almost exclusively studied *in vitro*. Here we discuss this intriguing possibility, its requirements in terms of electronic control of the implant, and the restrictions set by electrochemical safety limits, to form the basis for continued investigations *in vivo*.

Electro-activation is essential for triggering release but cannot be allowed to cause detrimental effects on the cellular microenvironment. The conducting polymer electrode is not analogous to a metallic electrode and direct currents can, and must, to some extent be tolerated to drive drugs out of the electrode. On the other hand, one must not overlook the possibility that by-products form in body fluids as a result of electro-activation, or that the neuronal circuitry is unintentionally activated, which means that potentials must be kept within strict boundaries. The solution that comes close at hand is to ensure that signals are maintained below the activation threshold. Then the question boils down to if this type of signal can be used to drive ionic drugs out of the electrode? The majority of studies focus on release by cyclic voltammetry (CV), a signal sufficiently slow to give room for diffusion limited processes to contribute, in contrast to fast stimulation pulses, generally designed to employ primarily capacitive effects.

Studies show that release can be precisely managed by the appropriate electrochemical driving signal. However, the means to keep exact control of charge and voltage are limited in an implant where three-electrode electrochemical systems are rarely implemented. Furthermore, the circuitry designed for stimulation *in vivo* cannot necessarily accommodate the same type of measurements and control as the electrochemical potentiostat. Therefore, electronics and implantable reference electrodes that meet this requirement need to be developed. If glial scarring is really to be treated by the suggested method it cannot come at the cost of connecting lab animals to fully functional external potentiostats but the solution must come as a miniaturized implant.

Finally we intend to outline the possibilities in terms of quantities of drugs that can be delivered and, to some extent, the variety of drugs that could come in question. Most studies focus on delivery of the anti-inflammatory drug Dex but results point out that other drugs with similar size and charge could also be potential candidates.

In summary, we present the possibilities of conducting polymer based release for glial scar treatment. Benefits of the method will be put in perspective with design challenges that have to be met from the electronics side. This information is essential for enabling more studies to proceed to the implant stage, shedding light on how to make the best out of this novel and exciting concept.

## Electrodeposition of conducting polymers

Conducting polymers can be deposited on top of microelectrodes using an aqueous electrodeposition process. The reaction is driven in a supporting electrolyte in which the monomer (M) is dissolved or dispersed together with appropriate counter ions (CI). The monomers oxidized at the surface of the working electrode build up an insoluble layer of conducting polymer on its surface. To maintain charge neutrality, the negatively charged CI are at the same time electrostatically entrapped in the formed material according to the following reaction:

(1) $$M+CI^{-}\overset{oxidize}{\to }{\left(M\right)}^{+}CI^{-}$$

The nature of the counter ion is decisive for the ionic exchange properties of the formed polymer (Bobacka et al., 2000; Jager et al., 2000). The CI can be small or large, inorganic or organic molecules, or a combination of several different negatively charged molecules can be included in the supporting electrolyte to form a more complex material. If a biologically relevant molecule is used as counter ion, the formed polymer can be biofunctionalized since the molecule is efficiently entrapped in the porous polymer matrix yet still available for reactions on the surface of the polymer material (Asplund et al., 2008). In addition it can be released from the polymer upon altering the polymer redox state.

There are some restrictions in the choice of biological ions that can come in question for the counter ion incorporation technique. The one step approach described above would work exclusively for negatively charged biomolecules. For the delivery functionality to be efficient, a further constraint is that the molecule must be sufficiently small to be able to diffuse through the polymer matrix. It is difficult to give a precise estimate on what could be considered as sufficiently small, since the porosity is not an absolute property but can be influenced by the electrodeposition process. To give some figure of merit, successful release has been shown for molecules up to the range of 0.5 kDa. Although some authors report release of substantially larger substances such as protein fragments, it is clear that this is more challenging and might primarily rely on actuation of the polymer material rather than electrostatic binding and release (Thompson et al., 2006; Evans et al., 2009; Richardson et al., 2009). This topic is discussed in detail in section Release Systems and Mechanisms.

As an alternative to the direct incorporation in Equation 1, an ion exchange approach can be used to drive ions into the already formed polymer film for subsequent release upon reversing the potential (Xiao et al., 2007). Furthermore, a carrier phase can be introduced into the material by allowing the polymer to form within a network of beads or fibers already containing the substance to be delivered (Abidian et al., 2006; Luo et al., 2011). A non-charged substance in the larger size range, which is made available in the supporting electrolyte, can be adsorbed to the surface in parallel with the deposition process and thereby also be mechanically entrapped although not electrostatically bound (Asplund et al., 2008). All these methods could in principle lead to a material with controlled delivery functionality similar to what is accomplished with the direct incorporation technique. The release dynamics can however be expected to differ. The focus of this review is primarily on controlled release based on direct incorporation according to Equation 1, although some results based on materials using other methods are also included.

## Dex delivery—quantities and efficiency

The theoretically possible inclusion of Dex in a polymer film, based on the counter ion incorporation technique, can be estimated by Equation 2 (Skotheim and Reynolds, 2006):

(2) $$m_{dopant}=\frac{Q_{dep}}{F}\cdot \frac{\gamma M_{dopant}}{2+\gamma }$$

*F* stands for Faraday's constant and equals 96485 C/mol and *Q_dep_* stands for deposition charge. With Dex as dopant, the commonly accepted assumption that the doping level γ = 0.3, and the molecular weight of Dex, *M_Dex_* = 392 g/mol, Dex inclusion per charge consumed in the electrodeposition process would be approximately 700 μg/C. The deposition charge density that would be considered reasonable varies, depending on the stability of the polymer system, but 300 mC/cm^2^ would clearly be within the realistic range. This would mean a total included Dex mass *M_Dex_*= 210 μg/cm^2^ according to Equation 2. Most likely higher deposition charges could be used (Li and Huang, 2007).

A handful of papers point out the Dex levels that would be required for efficient treatment of glial scarring. Based on the assumption that the volume of interest could be defined as a sphere of radius 500 μm enclosing the electrode (Wadhwa et al., 2006), and that an efficient concentration would be expected to lie within the range 0.2–1 μM (Golde et al., 2003; Shain et al., 2003; Zhong et al., 2005), further assuming a microelectrode radius of ~15 μm, the electrode would need to be able to deliver a concentration of 6–30 μg/cm^2^ from its surface.

Considering various papers report delivery of Dex in the range 3–126 μg/cm^2^ in total, several single efficient doses would be possible with the presented technology (Wadhwa et al., 2006; Moulton et al., 2008; Stevenson et al., 2010; Sirivisoot et al., 2011; Xiao et al., 2012). In light of these values one might argue that optimizing control is even more important than maximizing output per pulse or sweep.

## Release systems and mechanisms

**Figure 1:**
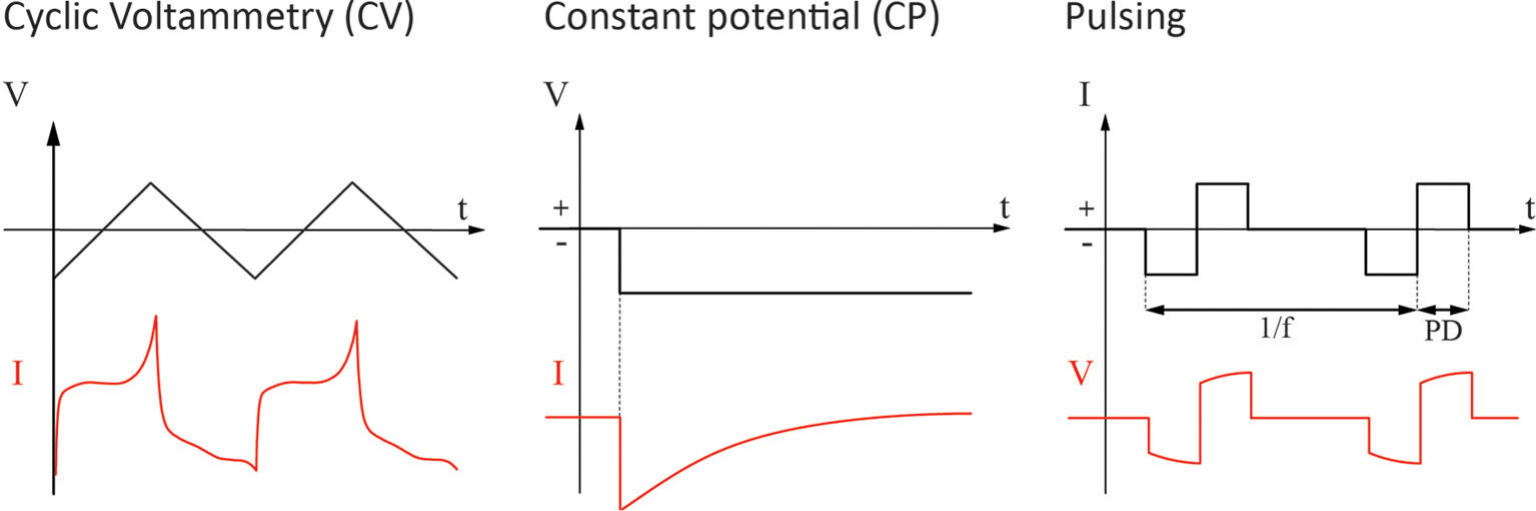
**The three different types of trigger signals for electrochemically controlled release that are discussed in the paper.** The driving signal is outlined in black and the follower signal in red.

The drug delivery from a conducting polymer is a result of the interplay between electrostatic interaction with the surrounding electrolyte, mechanical actuation of the film as a response to the different swelling states upon redox, and conformational changes in the polymer structure. To which extent each of the mechanisms contributes varies depending on the film morphology, the triggering signal and the use of any additional CI and must therefore be evaluated separately for each case. Three types of triggering signals are considered here namely redox sweeping, constant potential (CP) and pulsing (Figure 1). Redox sweeping is mainly referred to as the electrochemical measurement term CV.

The most simple release system would be a one-layer deposition where an anionic drug is included as a counter ion directly in the deposition process. Assuming that the drug is thereby homogenously distributed within the polymer film, and electrostatic interaction is the only responsible mechanism, the quantity of drug that is released should be proportional to the applied charge. This simplified view would imply that it is only the absolute charge transfer over the interface that matters for the quantity of drug delivered, regardless of if the charge is delivered as a pulse or a sweep. However, this is a crude simplification for a vastly more complex process (Kontturi et al., 1998; Majumdar et al., 2008). In fact, experimental data shows that the dynamics of the release signal greatly matters for the outcome.

In Table 1 a short compilation of release trigger signals and experienced results from different conducting polymer based release systems can be seen. Signals vary from steady potentials over slow CV sweeps to faster pulsed signals. From the experimental data reported in these studies it is evident that release is not solely ruled by charge transfer. Multiple ions contribute to charge transfer making the direct correlation of interfacial charge transfer to delivery of a specific species less trivial (Pyo et al., 1994; Jager et al., 2000; Pernaut and Reynolds, 2000; Li and Huang, 2007).

**Table 1:** **A compilation of experimental research concerning conducting polymer based drug delivery systems and the trigger signals used to control the release**.

**Study**	**Polymer:drug^a^**	**Delivery paradigm^b^**	**Detection scheme**	**Comment**
Pyo et al., 1994	PPy:ATP	CV: −1 V to 0 V at 10 mV/s CP: −0.4 V; −0.5 V	Electrochemical Quartz Crystal Microbalance (EQCM)/UV	CV more efficient than CP, −0.5 V CP sufficient for release but not −0.4 V
Pernaut and Reynolds, 2000	PPy:ATP	CV CP, −0.4 V; 0.7 V; 0.8 V	UV	CV more efficient than CP. No release for −0.3 V and increase of release rate from −0.4 to −0.8 V
Abidian et al., 2006	PEDOT:Dex, nanofiber template technique	1 V with a scan rate of 0.1 V/s for 10 s Comment see (^c^)	UV	−
Wadhwa et al., 2006	PPy:Dex	CV: −0.8 to 1.4 V at 100 mV/s	UV	CV more efficient than CP
Thompson et al., 2006	PPy:pTS:NT-3	Pulsed potential or current, −0.5 /+0.5 mA 5 Hz −20/+20 mA 5 Hz −0.6/+0.6 V 5 Hz CV: −0.8 to 1.0 V at 50 mV/s	Radiolabeling	−
Li and Huang, 2007	PPy:ATP	CV: 0 to −1.1 V at 10 mV/s Steps: −1.1 V, 60 s, 0 V, 60 s	Mass spectrometry	CV more efficient than a stepped potential
Evans et al., 2009	PPy:pTS:BDNF	Charged-balanced biphasic, 100 μs PD, 250 Hz, 60.2 mA, 25 μs open-circuit gap 3.78 ms short-circuit phase	Radio-labeling and ELISA	Comments see (^d^)
Ge et al., 2009	PPy:ATP or PPy:SSA + PPy:Cl block layer	CP: −0.3 V; −0.5 V; −0.8 V; and −1.0 V	Fluorometer and bioluminescence	−0.3 V sufficient for ATP release. Higher potential −0.8/−1 V = faster release
Luo and Cui, 2009a	PPy:Dex nanosponge, direct incorporation + sponge	CP: −2.0 V; −0.5 V, 5 s + 5 s at 0 V	UV and fluorometer	−0.5 V gave linear and steady release
Richardson et al., 2009	PPy:pTS:NT3	Charge-balanced biphasic, PD 100 μs, 250 Hz, stimulation currents 350−825 mA	Radiolabeling	
Leprince et al., 2010	PPy:Dex	CV: −0.8 to 0.9 V at 100 mV/s	UV	Scan rate influences release rate
Stevenson et al., 2010	PTTh:Dex	CP: 0 V; 0.6 V pulsed potential 0 V to +0.6 V at 1 Hz	UV	0.6 V CP is used to suppress NOT activate release
Ru et al., 2011	PPy:ATP	CP: −0.8 V	UV	
Li et al., 2012	PPy:TCF	CP: −0.3 V; −0.4 V; and −0.5 V vs. SCE	Gas chromatography mass spectrometry	−0.4 V vs. SCE gave efficient release
Xiao et al., 2012	PEDOT:Dex and PEDOT:Dex CNTs	CV: −0.8 to 1.4V at 50 mV/s	UV	

Results were selected with regard to on if they presented a system based at least partially on direct incorporation of drug at electropolymerization.

a Direct incorporation at electropolymerization unless otherwise stated.

b Potentials given are vs. Ag:AgCl unless otherwise stated.

c Not reported as CV, direct quote from paper “1V with a scan range of 0.1 Vs^-1^ for 10 s.”

d Should be noted that there was no dramatic difference between active and passive release.

Abbreviations: pTS, paratoluenesulphonate; NT-3, neurotrophin-3; BDNF, Brain Derived Neurotrophic Factor; SSA, sulfosalicylic acid; TCF, trichlorfon; CNTs, Carbon NanoTubes.

Some early work on PPy:ATP membranes gives important insight into the different mechanisms responsible for CP based drug delivery (Pyo et al., 1994; Pernaut and Reynolds, 2000). Based on parametric studies on inclusion and release of Adenosin Triphosphate (ATP), it was found that small, highly mobile cations are initially driven into the film and first after several minutes the release of ATP becomes the dominant process. Furthermore, even though the dissociation of the ionic drug from the polypyrrole chain may be fast, the actual release from the film is driven by diffusion and is therefore a slow process (Pernaut and Reynolds, 2000; Wadhwa et al., 2006; Li and Huang, 2007; Leprince et al., 2010). Fast signals would according to these findings mainly result in exchange of small anions/cations at the superficial layer of the polymer, for which the diffusion coefficients are low.

The majority of studies report that CV is vastly more efficient for driving release than CPs (Pyo et al., 1994; Pernaut and Reynolds, 2000; Wadhwa et al., 2006; Li and Huang, 2007). This underlines the complexity of the events involved in the release process far beyond what can be accounted for by the simplified electrostatic equation. The structural changes in the polymer upon redox are expected to play a major part. Most likely all the stored drug is not immediately accessible for release. Diffusion of ions within the film, and rearrangement of the polymer chains over time, exposes new deposits of drug that were not immediately accessible for the first release attempt.

Electro-actuation contributes to such conformational changes but in addition is expected to serve as a purely mechanical release mechanism for any substance entrapped within the film (Abidian et al., 2006; Thompson et al., 2006; Li and Huang, 2007). The polymer film can both shrink and swell upon reduction depending on the size of the counter ion (Jager et al., 2000). For CI in the intermediate size range such as Dex and ATP it is not straight forward to predict which mechanism would dominate. Nevertheless, repeated redox cycling will swell and shrink the film interchangeably. Actively reversing the potential at a slow rate, such as in CV, contributes to more efficient release for the anodic cycle both through inner rearrangement and through mechanical contraction of the matrix.

In summary, the efficiency with which interfacial charge transfer can contribute to release of the intended drug depends on the dynamics of the trigger signal. Furthermore, depending on diffusivity and thickness of the individual polymer film, the output of drug can be expected to vary. It is possible to alter the electrodeposition protocol to achieve higher diffusivity of the film, or even build layers with different properties to optimize the active vs. passive release behavior for matching a certain release protocol (Pernaut and Reynolds, 2000; Ge et al., 2009; Ru et al., 2011; Jiang et al., 2013). Moreover, the effect of the purely mechanical actuation can be further exploited by including pores in the film, for instance by a templated electrodeposition process (Abidian et al., 2006; Luo and Cui, 2009a; Luo et al., 2011). While in early work, the systems studied were single layers, advanced strategies to increase porosity of the films and boost their storage capacity are more frequently reported in recent times. In a system where entrapment is not solely based on electrostatic interaction, the mechanical actuation could be expected to be the dominant mechanism for release.

Despite the complexity of delivery mechanisms, one can make a few general assumptions on which type of signal that would be the most efficient for release from a polymer system regardless of its structure. It is clear that the theoretical arguments speak in favor for slow signals rather than fast pulsing, and especially for CV which would allow both mechanical actuation and electrostatics to contribute. There is a substantial risk that fast signals will not lead to efficient transfer of the intended ions. Furthermore, for a pulsed signal, the electrode will return to the open cell potential in between releases (mono-phasic pulsing) or even be actively reversed (bi-phasic pulsing). This means each delivery will be immediately followed by a signal actively driving the ionic flow in the opposite direction. This could in theory mean a reuptake of drug. However, the infiltration of small ions from solution to replace the larger ions delivered is more likely. One way to minimize the plausible reuptake of drug would be to allow the molecules additional time to diffuse away from the surface and be replaced by other ions at the reversed potential, speaking for the introduction of an interpulse delay time.

It should be noted that despite all these points speaking for active release with slow signals, release has been experimentally proven also for systems with fast pulsing (Evans et al., 2009; Richardson et al., 2009; Thompson et al., 2010). Thompson et al. do not specifically report pulse duration, only that they use biphasic stimulation delivered at a 5 Hz frequency. Granted that this means the full time was used for delivery of pulse trains this would however mean PDs in the range of 100 ms which is far from the PDs that would normally be used to trigger neural activity. The pulses reported by Evans et al. and also by Richardsson et al. come closer to real stimulation parameters with pulse widths of 100 μs delivered at 250 Hz (Evans et al., 2009; Richardson et al., 2009). It should be mentioned that there was no dramatic difference between active and passive release in the PPy/pTS/BDNF system but clearly an effect for the PPy:pTS:NT3 system. This would speak for a release controlled by conformational changes of the polymer rather than an electrostatic driving force. The authors in addition argue for that changes in hydrophobic properties of the polymer matrix would contribute to release in this case.

The CV ranges reported vary widely. The lowest vertex potential reported is at −1 V and the highest vertex potential at 1.4 V vs. Ag:AgCl. Commonly, only the anodic part of the sweep is used. It has also been pointed out that the scan rate further influences release efficiency of the single CV (Leprince et al., 2010). Sweep rates reported are in the range of 10–100 mV/s. For the constant potential driven systems, the potentials required to actively drive release are in the range of −0.3 to −0.5 V vs. Ag:AgCl. Clearly this will depend on the nature of the ionic drug, which conducting polymer that is used and the morphological properties of the individual polymer layer.

To some extent, a conducting polymer film that is physically degrading upon electrochemical stress can also act as a controlled delivery system. As the film falls apart, molecules that were immobilized in the structure are released at a rate that can be controlled by the level of stress. The weakest link is often the adhesion to the underlying substrate leading to complete or fragmental delamination, depending on the film cohesion. It might be difficult to judge whether a certain system performs delivery based on actuation, electrostatic interactions or simply by degradation, something that might be less favorable with an electrode intended for long term use, by only studying the release of the intended species. Ideally, one should therefore analyze if other molecules or particles are expelled from the film in parallel to the drug (Boehler and Asplund, in press). It should also be noted that conducting polymers degrade by over-oxidation meaning that the electronic structure is disrupted leading to a non-conducting material. PPy is more vulnerable to over-oxidation than PEDOT, and also the counter ion has an influence on the electronic stability (Yamato et al., 1995; Thaning et al., 2010). For this type of degradation the material itself remains at the electrode but progressive over-oxidation will influence the ion conducting properties and thereby the delivery mechanism over time.

## Release trigger signals—from the side of biological safety

In the previous section, the efficiency of the three release signals was discussed. However, other practical aspects such as the availability of devices capable of delivering these signals, as well as the safety for the biological environment, should naturally be taken into consideration. In the following section, the three trigger signals in Figure 1 are therefore discussed with regard to the safety of neurons.

In general, the restrictions that would apply to a signal to be applied *in vivo* could be summarized as follows:

1. The signal transfer should take place through reversible processes which do not lead to the formation of electrochemical by-products in the tissue or corrosive reactions at the electrode.
2. The signal must not trigger undesired activity in the neural network.
3. The signal must not induce damage to neurons.

These conditions do not completely apply for the situation where the aim is to use the signal to drive controlled release. In this case, ideally the trigger signal should be designed to be practically invisible from the side of the neuron, yet still be efficient for pushing out drugs in a reasonable time frame and with good control of the delivered amount. While restriction 1 is of the utmost importance for metallic electrodes it is not directly transferrable to polymer electrodes. The aim is to exchange the ionic drug in the polymer for other ions naturally available in tissue so irreversibility is to some extent here a necessity. However, the limitation of the electrode potential to prevent redox reactions in proteins and water as well as pH-shifts is a must.

It is also highly desirable that the signal used for drug release does not result in undefined excitation of surrounding neurons as in restriction 2. Normally, when evaluating how to stimulate neural tissue, the ambition is to assemble signals that are efficient in precise activation of a defined population of cells at current/pulse durations chosen to induce minimal electrochemical stress on the electrode. For the drug release approach, the signal needs to be optimized from close to the opposite perspective. The ambition would be to use a release signal that does not substantially influence surrounding cells but is efficient in pushing drugs out of the electrode.

Last, but not least, it is well-known that electrical stimulation of neurons can induce cellular injury even when the delivered stimulation is well within the boundaries given by the electrochemical safety (Shannon, 1992; Veraart et al., 2004). Normally such effects do not occur below the levels which can trigger neural activity which would ensure that a signal that confines to condition 2 is a conservative limit also for restriction 3. However, these injury thresholds are most of the time given with consideration to the pulsing parameters that would be considered normal for neural excitation. It is therefore advisable to take a closer look at the safety boundaries from this perspective when more unconventional pulsing parameters might be put into use.

### Pulsing

**Figure 2:**
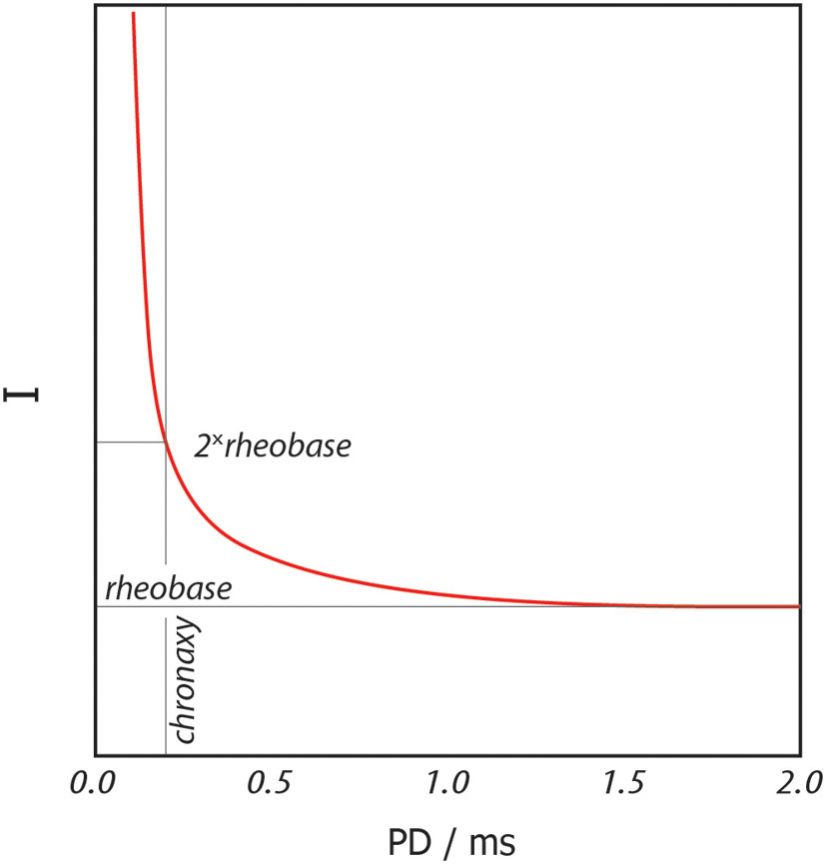
**Strength-duration relationship for excitable tissue.** Red line shows the threshold current, *I_th_*, at which neuronal tissue is excited for a rectangular stimulation pulse of duration PD.

A standard pulse for recruitment of intracortical neurons would be a biphasic train of rectangular pulses of constant current delivered at a frequency in the range of 100–400 Hz, commonly around 200 Hz (see Figure 1). The minimum current strength needed to excite neuronal tissue (threshold current, *I_th_*) depends on the pulse duration (PD) as in the strength-duration relationship depicted in Figure 2. PD should as a rule of thumb be chosen close to the chronaxy of the intended target tissue which in practice means durations in the range of 100–300 μs for myelinated nerve fibers. Longer pulse durations would not be energy efficient for activation of neurons and in addition would increase the probability for non-reversible corrosive processes at the electrode site (Tehovnik, 1996).

When stimulating at PDs close to the chronaxy, typical threshold currents for neural activation would be estimated to be around 10 μA, but recent studies show that actual activation thresholds can be expected to be even lower than 4 μA (reported for 200 μs PD and 250 Hz) (Tehovnik et al., 2006; Histed et al., 2009). Since the objective of the PEDOT electrode is drug delivery rather than activation, stimulation should be kept below threshold not to evoke neural activity unintentionally. In practice and according to the generally accepted current-duration relationship, this leaves two alternatives (Tehovnik, 1996). Either shortening the pulses to increase the threshold current, or use longer pulses and stay well-below the rheobase. For a pulse substantially longer than the chronaxy this means at least below 2 μA, preferably even lower. The longer the pulse, the more charge would be expected to be required to activate neurons. This clearly rather speaks for maximizing charge and thereby possibilities for ionic delivery by driving longer pulses at lower currents.

If a small and highly local amount of activated neurons can be tolerated, currents can naturally be set higher. This is something that would have to be evaluated with respect to the exact placement of the particular electrode. With knowledge of the excitability constant, *K*, of the target tissue, efficient spread of stimulation can be estimated, over the radius *r*, for a given case according to *I* = K*r^2^ (Tehovnik, 1996; Tehovnik et al., 2006). This would provide a reasonable estimate for the volume of cells affected by a pulse if exceeding the threshold in the given case.

Tissue damage can occur as a consequence of inappropriately chosen stimulation parameters, even though the given stimulation is within the electrochemical safety limits of the electrode material. It is broadly accepted that important factors to consider are charge per phase and charge density delivered from the electrode (McCreery et al., 1990; Shannon, 1992). The mechanisms behind such effects are not completely understood but it is believed that electroporation is a main contributor to this kind of tissue damage as well as mass action phenomena, for instance depletion of oxygen or glucose, or excessive release of glutamate, caused by local neural overactivity (Merrill et al., 2005). Since signals in the case discussed here should be chosen with the aim not to induce neural signaling, only electroporation would need to be considered.

Taking also restriction 3 into account, there is reason to ask if there is a risk for the occurrence of electroporation based on this type of pulsing? A detailed insight into how the threshold for electroporation can be expected to vary with other stimulation parameters is given by Butterwick et al. (2007). One conclusion of their work is that, for longer pulses (PDs in the range of ms), the threshold current density at which electroporation can be expected to occur is lower than for shorter pulses. The threshold current density scales with PD approximately as 1/sqrt(PD). It is also clear from their results that with pulsing frequencies exceeding 50 Hz repetition frequency thresholds are significantly lower than for single shots. For electrodes smaller than 300 μm it is further concluded that total current would be the main limitation, and injury thresholds would not to the same extent be influenced by current density as is seen with the larger electrodes. Their results are based on measurements in chicken retinas but correlate well to measurements in earlier work concerning injury thresholds in cortical tissue (McCreery et al., 1990; Shannon, 1992; Veraart et al., 2004). It is therefore assumed that the safe limits reported would be a reasonable figure of merit for stimulation of any target tissue in the central as well as in the peripheral nervous system.

The lowest threshold where stimulation induced damage was detected in their experiments was determined to be 61 mA/cm^2^ at a pulse duration of 6 ms for repetitive pulsing (>50 Hz). Translated to a microelectrode of 50 μm that would correspond to a charge per pulse of 7.2 nC. However, for pulses shorter than the ms, threshold currents are substantially higher. Deduced from the data presented by Butterwick, for the 600 μs, expected thresholds would rather be in the range of 0.2 A/cm^2^. Due to the shorter pulse length this would in fact mean a lower charge per phase threshold, for the 50 μm electrode 2.3 nC/phase. This would speak for that electroporation could occur even with a minor elevation of the stimulation current.

The study also presents results regarding the influence of the size of the electrode and the values cited above relate to a large electrode, not a microelectrode. If taking their results concerning microelectrodes into account, here smaller than 300 μm in diameter, the threshold charge per phase that could safely be delivered did in fact not depend on electrode size. The smallest electrode used in their experiment was ~100 μm in diameter and the threshold, when investigated on the retina cells using 600 μs single pulses, were found to be ~70 nC per phase. This threshold should, according to their theory of size independence, also be valid for smaller electrodes. Even though these particular results describe thresholds for one single pulse, there is substantial margin to the 1.2 nC given by the 2 μA and 600 μs suggested as suitable sub-threshold stimulation (second paragraph, section Pulsing).

In summary, also from the perspective of restriction 3, it would be recommendable to work with PDs longer than the chronaxy. It further appears as if PDs are on the longer side, and threshold current is estimated to be below the rheobase, there is no substantial risk for disruption of cellular membrane integrity as a consequence of the pulsed trigger signal.

In order to establish boundaries for safety it is of interest to glance at the field of deep brain stimulation (DBS), where intracortical stimulation has been therapeutically used over more than a decade. The technology is FDA approved and one could thereby argue that these stimulation protocols have already been proven safe for use in patients. Kern and Kumar (2007) report common values for the Sub Thalamic Nucleus (STN) DBS to be in the range 130–185 Hz with typical pulse durations of 60 μs. However, the extremely short pulse durations used for DBS are most likely not efficient for drug expulsion. Release relies to large extent on slower processes as has already been discussed in the previous section.

### Cyclic voltammetry

It has already been pointed out that slow redox sweeping of the electrode has been found efficient for delivery of ionic drugs. The stimulation effects of CV in brain tissue are not specifically addressed in the literature. Theoretically, if the current-duration curves are extrapolated into infinitely long pulses, which would be a reasonable approximation for the smooth transitions of a CV curve, the suggestion would be that as long as the current is still below the rheobase the neurons would not be excited.

If the neuronal activity is not directly influenced, the restrictions would rather be set by the possibly detrimental electrochemical reactions that could occur as a by-product of any excessive voltage. The two parameters to consider are the vertex potentials and the sweep rate. The non-conservative constraints for the vertex potentials would be given by the water window (−0.6 V to 0.8 V vs. Ag:AgCl) since the evolution of hydrogen (−0.6 V) and oxygen (0.8 V), respectively, would occur at the electrode upon exceeding this window. Over-oxidation of PEDOT would not be expected to occur at voltages lower than 1.1 V and is therefore not a limiting factor in this case. This is however not sufficient to support that no other electrochemical reactions of importance take place within this window. Furthermore, appropriate reference electrodes are often not available in the implanted situation meaning that possible variations in applied voltages must be taken into account. Materials do either not deliver a stable potential in chronic implantations or biofouling clogs pores in ion-selective membranes.

One example on actual parameters used in a similar biological setting can be found in recent work monitoring electrochemical characteristics of implanted electrodes. Kane et al. (2011) employs the full water window at a sweep rate of 50 mV/s for their *in vivo* CV investigations and do not report any adverse tissue response as a consequence. In the sensor literature, fast CV is often suggested for instance for *in vivo* monitoring of neurotransmitters. It should be noted that so far sweep rates used are in the range of 300 V/s and thus far beyond what has been proposed for drug release (Pihel et al., 1996). The biological response was not the primary target for the analysis in either study and was therefore not carefully observed. Scans used for analytical purposes in an experiment with a sedated animal, and limited to a few occasions, are not necessarily suitable for repeated use in proximity of a population of highly sensitive cells. In both studies animals were anaesthetized which might not be possible to perform on a bi-weekly to weekly basis needed to support efficient drug release.

### Bias potential

A bias potential of approximately −0.5 V vs. Ag:AgCl could be used to drive drug release. Cells are not expected to respond adversely to a steady potential if kept within reasonable boundaries. For instance, a positive interpulse bias potential of 0.6 V has been suggested as a method for boosting performance of iridium oxide electrodes and has been applied to SIROF coatings *in vivo* (Cogan et al., 2006; Kane et al., 2011). The authors comment that the long-term consequences of the low net current needed to maintain the bias potential has not yet been explored. However, for the drug release, the bias potential would only be applied at the specific occasions where drug release is requested which qualifies as a limited time frame and should be safe for use.

## Discussion

### Other release strategies and drugs

Apart from Dex, also various other anti-inflammatory drugs like α-MSH, IL-1-RA, or heparin have been proposed in literature for treatment of inflammatory reactions. These molecules target different receptor pathways in the inflammation cascade and can thus be interesting alternatives or complements to the Dex (Bridges and Garcia, 2008; Go et al., 2012). All of the listed molecules are typically delivered from passively eluting systems due to their chemical properties and overall size, which makes them incompatible with the ion-exchange principle provided by conducting polymers. In contrast, Dex is in the appropriate size range (392 Da) and features an anionic charge characteristic, which makes is eligible for both passive and actively controlled release systems. Furthermore, Dex is known to be the most potent glucocorticoid drug for anti-inflammatory treatment due to its effect in multiple receptor pathways, which reveals this drug as first choice for the realization of an anti-inflammatory drug eluting system for treatment of the glial scar. For a more detailed description of relevant molecules and their impact on the adverse host response to implanted biomedical devices the excellent review by Bridges et al. is strongly recommended (Bridges and Garcia, 2008). It should be mentioned that it would be possible to use a deposition technique not relying on direct incorporation to combine the active delivery approach also with these substances (Abidian et al., 2006; Luo et al., 2011).

It is a valid question to ask how the actively controlled release of Dex from a microelectrode would compare to a delivery coating passively leaking Dex into tissue, or systemic treatment (Shain et al., 2003; Spataro et al., 2005; Zhong and Bellamkonda, 2007). The latter has the clear downside of requiring much higher total doses distributed to reach an efficient concentration at the point of interest and side-effects would thus be a main concern. In situ delivery, avoiding the counterproductive neuroendocrine feedbacks, is from this perspective clearly beneficial regardless of if the system is based on a passive or active release approach.

The active release system offers controllability but for a limited quantity whereas the passive system can cover the complete surface of a probe, thereby storing a much larger quantity, but hold few options to influence release dynamics. From the data available in literature it is difficult to judge whether such a system would be more successful in mitigating inflammation than the actively triggered release approach. The answer to this question could only be given if the necessary time course of the treatment was known. It is still an open question whether the anti-inflammatory treatment over the time course of a month, which would be the critical period in which the scar forms, influences the state of the surrounding tissue also in the long term (Szarowski et al., 2003; Biran et al., 2005; Potter et al., 2012). If it does, the lower quantity delivered by the microelectrodes might still be sufficient, meaning higher drug load is not a substantial benefit but could be traded for more precise release control. On the other hand, treatment might be needed for a much longer time period which would make the opportunity to load a higher quantity of drug much more attractive. A certain control of release dynamics in the passive release system could still be accomplished by carefully tailoring the degradability properties of the release matrix. In theory, nothing speaks against that such a system in the future could include certain responsiveness to biological factors, for instance react to chemical factors present for a higher degree of inflammation, by increasing the release rate.

It should be noted that such a delivery matrix, which can act as the golden standard for passive release, does not yet exist. Thus, the passive release system relies on substantial development in this direction in the same way as the polymer electrodes hold room for future optimization. Recent result show that also surface immobilized Dex might have an effect for improving probe integration which illustrates the need for further work to elucidate the underlying mechanisms involved in the tissue response to Dex (Grand et al., 2010). In summary, the passive delivery approach should be pursued in parallel with the active delivery systems, as an interesting alternative or even additional approach, since the use of the one delivery system in fact does not exclude the use of the other as a complement.

### Release trigger signals—concluding remarks

Taking all the considerations regarding efficiency and safety into account they all speak in favor for slow release signals. CV seems to be the most suitable from the efficiency perspective and most likely a moderate use of voltammetric sweeping will not be harmful to tissue or lead to undeliberate excitation of the neuronal network. This however needs to be confirmed in experiments.

In practice, the equipment needed to perform well-controlled CV at the implant site might however not always be available. Proper CV measurements require a three-electrode electro-chemical control system and the technologies for on-probe integration of stable reference electrodes on implants are not yet established (Tolosa et al., 2013).

Considering this practical aspect, it might therefore still be the most convenient to use a pulsed signal for release rather than a sweep. In this case it would be advisable to aim for low currents and PDs significantly longer than the chronaxy. Furthermore, to present as little stress as possible to the tissue, low pulsing frequencies should be used. Lowering the pulsing frequency and compensating by extending the overall stimulation time should have little effect on the release itself but would allow higher currents before stimulation induced damage to neurons can be expected to occur (Butterwick et al., 2007). Charge balance is not a necessity for this kind of drug release. On one hand it might contribute to increased actuation, on the other hand it might be counterproductive if the Dex ions are immediately retrieved by the reversed pulse. An interpulse delay could mitigate the second effect.

**Figure 3:**
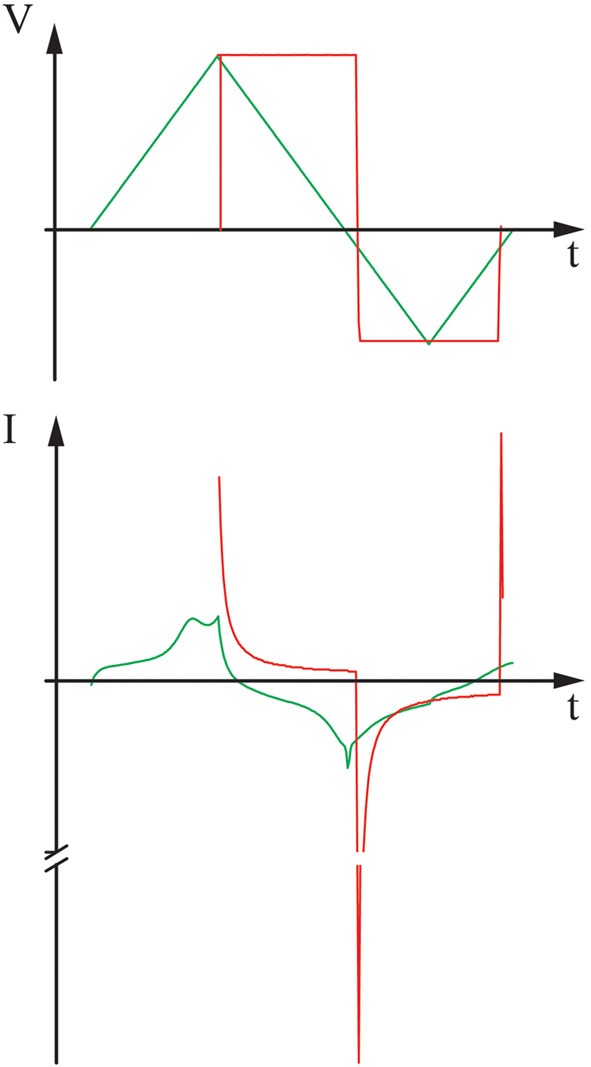
**A comparison between the current response to a swept voltage vs. a stepped voltage for a PEDOT:Dex delivery system.** Measurement was performed in PBS and vs. an Ag:AgCl reference.

One possibility to, with a simple device, imitate the effect of a CV is to exchange the sweep with interchangeable voltage steps, set to the upper and lower vertex potential. Microsized devices capable of maintaining a fixed voltage vs. a reference, and suitable for implantation, have been described by others (Islam et al., 2010). In Figure 3, the current response of a PEDOT:Dex electrode to a cyclic voltammogram can be seen as well as the current response to a rectangular pulse. It is clear that the current flow follows another scheme than in the carefully controlled slow CV. What this in practice would mean for the drug release should be further investigated experimentally.

Finally, not only efficiency of release but also controllability should be considered. For the application, it is desirable that a precise dose of Dex can be delivered at each attempt, which means the response to the trigger signal must be predictable. Taking the complexity of events involved in release into account, such one to one correlation is not trivial to accomplish. More likely, the response of the polymer will change over time and with each delivery attempt, something that must be carefully considered to ensure the trigger signal has the intended effect throughout the full experimental time frame. This needs to be further investigated before a specific polymer delivery system is put into use.

Since the drug delivery systems reviewed here are intended for local treatment targeting only the cells in the immediate vicinity of the electrode, it is highly desirable that the very same electrode used for delivery can still function as a neural interface. For recording this is not expected to be a problem since both PEDOT and PPy are known for their low impedance in this respect. Although the drug delivery electrodes in general are outperformed by the regular surfactant based conducting polymer materials in terms of impedance, it has repeatedly been shown that also drug containing conducting polymer electrodes still lower the impedance in comparison to a bare metallic surface (Wadhwa et al., 2006; Luo et al., 2011).

For stimulation the central question would be if stimulation can be performed without unintentionally triggering release? From the literature reviewed here it is not possible to make a conclusive statement on whether this would at all be possible. As discussed, some authors do indeed present release based on the type of fast pulsing that would be common to use for stimulation purposes (Evans et al., 2009; Richardson et al., 2009). However, it can be concluded that slow sweeps are vastly more effective for drug release (Table 1) and several authors report that they need to reach a certain voltage before actual release is triggered (e.g., Pyo et al., 1994; Pernaut and Reynolds, 2000). It is plausible that smart materials design can further exploit this effect making the release-free stimulation possible within certain boundaries.

### Conflict of interest statement

The authors declare that the research was conducted in the absence of any commercial or financial relationships that could be construed as a potential conflict of interest.
